# Association of blood pressure and hypertension between parents and offspring: The Korea National Health and Nutrition Examination Survey

**DOI:** 10.1038/s41440-022-01089-7

**Published:** 2022-12-02

**Authors:** Seoyun Jang, Susan Taejung Kim, Yun-Kyung Kim, Young Hwan Song

**Affiliations:** 1 Department of Pediatrics, Seoul National University Children’s Hospital, Seoul, the Republic of Korea; 2 Department of Pediatrics, Korea University Ansan Hospital, Korea University College of Medicine, Ansan, the Republic of Korea; 3 Department of Pediatrics, Seoul National University Bundang Hospital, Seongnam, the Republic of Korea

**Keywords:** Hypertension, Blood pressure, Adolescent, Cardiovascular diseases

## Abstract

As the number of hypertension cases in the pediatric population is growing, we aimed to investigate the parent-offspring association of hypertension in Korea. We performed a cross-sectional analysis using the data of children and adolescents aged 10–18 years and their parents extracted from the Korea National Health and Nutrition Examination Survey (2008–2018). We analyzed the correlation of blood pressure (BP) between offspring and their parents and investigated the odds ratio (OR) of having hypertension in offspring based on parental hypertensive status. A total of 3996 children and adolescents (2224 boys and 1772 girls) aged 10–18 years and their parents (3197 fathers and 3197 mothers) were evaluated. Both boys and girls had positive associations with both parents for systolic and diastolic BP. When neither parent, only the father, only the mother, or both parents were hypertensive, 6.6%, 10.4%, 13.3%, and 25.3% of boys and 6%, 12%, 12.7%, and 22.1% of girls had hypertension, respectively. The risk of having hypertension among offspring was approximately two times higher when one parent was hypertensive and over four times higher when both parents were hypertensive compared to that among controls whose parents were not hypertensive (OR: 2.230, 1.655, and 5.021 in boys with hypertension and 2.321, 2.169, and 4.554 in girls with hypertension in the mother only, the father only, and both parents, respectively). We identified familial aggregation of hypertension in Korea. As there was an increased likelihood of having hypertension in children with parental hypertension, parental hypertension may be utilized as a screening tool for hypertension in children.

## Abstract (web summary)

We investigated the familial association of hypertension between offspring and their parents in South Korea. The prevalence of hypertension in boys and girls tended to increase based on the number of parents with hypertension. The risk of having hypertension among offspring was approximately two times higher when one parent was hypertensive and over four times higher when both parents were hypertensive compared to controls whose parents were not hypertensive. OR odds ratio, HTN hypertension, BP blood pressure.

## Graphical abstract

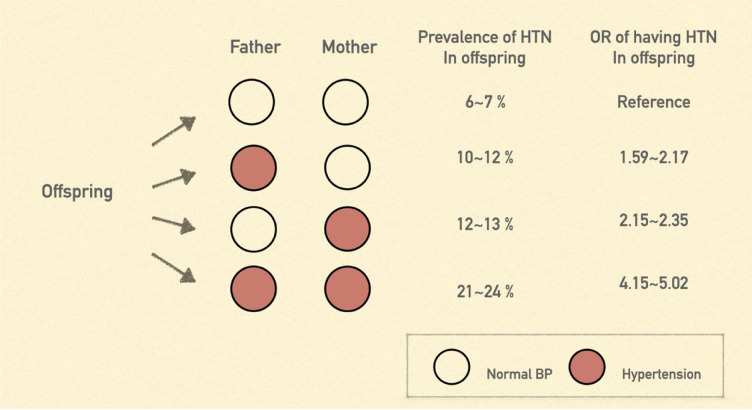

## Introduction

Hypertension causes serious medical problems in adults and has been found to be predictive of cardiovascular mortality and morbidity [1, 2]. As hypertension is a complex disease affected by genetic susceptibility, environmental factors, and their interactions, clearly identifying the causative genes for hypertension has been challenging [3, 4].

In recent decades, many studies have demonstrated the influence of genetics on hypertension and the familial association of blood pressure (BP) in various populations [5–8]. Previous studies have reported a significant increase in BP and the prevalence of hypertension in children and adolescents [9]. As demonstrated, children and adolescents with higher BP are at increased risk of developing hypertension and cardiovascular disease later in life [10], and prevention and early intervention are crucial for children with elevated BP. Therefore, identifying familial associations of hypertension between parents and their offspring would enable us to stratify the relative risk of elevated BP in children and adolescents, leading to the prevention and early management of cardiovascular disease. However, despite its clinical importance, there are few population-based studies on the familial association of hypertension, particularly in Korea.

The present study investigated the familial associations of hypertension between children and adolescents and their parents in Korea. We also analyzed the relative risk of developing hypertension in children and adolescents with hypertensive parents.

Point of View

- Clinical relevance
  The association of blood pressure between offspring and their parents may help predict the relative risk of hypertension in children and adolescents with parental hypertension.
- Future direction
  Further studies are needed to discover the genes causing hypertension.
- Consideration for the Asian population
  We aimed to help predict hypertension in other Asian countries by providing data on the Korean familial association of hypertension.

## Methods

### Study population

This study was based on data acquired from the KNHANES, a nationwide cross-sectional survey performed annually by the Korea Centers for Disease Control and Prevention (KCDC). The annual survey includes physical examinations, health and medical interviews including information on physical activity and antihypertensive medication use, nutritional evaluations including total calorie intake and salt intake data, and laboratory measurements. Trained public health investigators conduct the survey. A detailed description of the plan and operation of the survey is available on the KNHANES website [11].

In the present study, KNHANES data from 2008–2018 were assessed, and participants who were 10–18 years of age at the time of the survey and their parents were included. Only the offspring whose parents participated in the survey were included. Offspring and parents who did not have data for anthropometric measurements, BP values, lipid profile values, glucose levels, physical activity, total calorie intake, or salt intake were excluded. Among the study subjects, none had congenital heart diseases or renal failure at the time of the survey.

The KNHANES protocol was approved by the Institutional Review Board of Seoul National University Bundang Hospital (IRB No. X-1604-344-901), and informed consent was obtained from all participants.

### Anthropometric measurements

Anthropometric measurements were taken using standard techniques by trained personnel according to the KNHANES instructions. Height was measured to the nearest 0.1 cm using a vertical stadiometer (Seca 225; Seca, Hamburg, Germany). Weight was measured to the nearest 0.1 kg using an electronic scale (L-6000-20; G-tech, Seoul, Korea). Body mass index (BMI) was calculated as weight (kg)/height (m^2^). According to the American Heart Association (AHA) guidelines, BP measurements were performed by manual auscultation with a mercury sphygmomanometer (Baumanometer Desk Model 0320, America, 2008–2012, Baumanometer Wall unit 33, America 2013–2018). Appropriately sized cuffs were selected according to the estimated circumference of the participant’s upper arm. After 5 min of rest, BP was measured in a sitting position with the right forearm placed horizontally on the table. An appropriately sized cuff was placed above the brachial artery and inflated to the point where the pulse could no longer be detected. The cuff was deflated at a rate of 2 mmHg per second. The appearance of sound (Korotkoff phase I) was defined as the systolic BP (SBP), and the loss of all sound (Korotkoff phase V) was defined as the diastolic BP (DBP). Three separate measurements were taken 1–2 mins apart.

### Diagnosis of hypertension

For subjects aged <16 years, hypertension was diagnosed when the average of the three separate measurements of SBP and/or DBP was above the 95% percentile, adjusted for sex, age, and height [12], using the BP reference values for Korean children and adolescents [13]. For subjects aged ≥16 years, hypertension was defined as an average SBP value ≥140 mmHg and/or an average DBP value ≥90 mmHg [14]. Additionally, all the subjects taking antihypertensive medications were classified as having hypertension, regardless of their BP values.

### Laboratory measurements

Blood samples were obtained from all the subjects participating in this study in the morning after an overnight fast and were analyzed in the national central laboratory. Serum total cholesterol, low-density lipoprotein cholesterol, high-density lipoprotein cholesterol, triglyceride, and plasma fasting glucose levels were measured.

### Statistical analysis

SPSS version 23 (SPSS Inc., Chicago, IL, USA) was used for statistical analysis. Analysis of covariance was used to compare the mean weight, height, and BMI adjusting for age; SBP and DBP were adjusted for age and height and compared between boys and girls. When comparing these variables between fathers and mothers, the means of the variables were used without adjustment. Sex-, age-, and height-specific z scores of SBP and DBP were used as the BP values for children and adolescents, using the Korean reference BP value data [13].

To account for differences based on sex, comparative analyses were performed separately for boys and girls and fathers and mothers. Linear regression analysis was used to evaluate the correlation between offspring BP z scores and parental BP. For linear regression analysis, we excluded the subjects taking antihypertensive medications. We estimated multivariable-adjusted prevalences of hypertension by logistic regression analysis using R statistical programming language. Using logistic regression analysis, we estimated the odds ratio (OR) of having hypertension in offspring based on parental hypertension and compared the group with parental hypertension with the control group, where neither parent was hypertensive. To investigate the relationship of BP between offspring and their parents, linear regression analyses were performed adjusting for age in Model 1 and adjusting for BP-influencing factors, including the BMI, lipid profile value, glucose level, physical activity, total calorie intake, and salt intake of offspring, in Model 2. For offspring, the BP z scores were used as the BP values in these analyses because the measurements of BP varied according to sex, age, and height in youth. As the KNHANES followed a multistage clustered probability design for the sampling plan, we applied sample weights for the analysis. Two-tailed *P value*s less than 0.05 were considered statistically significant.

## Results

A total of 3996 children and adolescents (2224 boys and 1772 girls) and their parents (3197 fathers and 3197 mothers) were evaluated. As siblings were included in the study population, the total number of parents was less than that of children and adolescents. Table 1 shows the characteristics of the offspring and their parents. Weight, height, BMI, SBP, the prevalence of hypertension, triglyceride levels, fasting glucose levels, energy intake, salt intake, and physical activity were higher in boys than in girls. Total cholesterol, HDL cholesterol, and LDL cholesterol levels were more elevated in girls than in boys. There was no statistically significant difference in age or DBP between boys and girls. In comparing their parents, age, weight, height, BMI, SBP, DBP, hypertension prevalence, total cholesterol level, triglyceride level, fasting glucose level, energy intake, salt intake, and exercise time were higher in fathers than in mothers. Similar to the results of their offspring, HDL and LDL cholesterol levels were more elevated in mothers than fathers.

**Table 1:** Characteristics of subjects

	Boys	Girls	*p* value*	father	mother	*p* value**
Number	2224	1772		3197	3197	
Age (year)	13.7 ± 2.5 (13.7, 0.1)	13.7 ± 2.6 (13.7, 0.1)	0.316	45.6 ± 4.9	42.7 ± 4.7	<0.001
Weight (kg)^a^	56.6 ± 15.8 (56.5, 0.2)	49.7 ± 11.5 (49.7, 0.2)	<0.001	72.3 ± 10.4	53.3 ± 14.1	<0.001
Height (cm)^a^	162.8 ± 13.0 (163.0, 0.1)	156.4 ± 8.5 (156.4, 0.1)	<0.001	171.3 ± 5.9	158.9 ± 5.4	<0.001
BMI (kg/m^2^)^a^	21.0 ± 4.0 (20.9, 0.1)	20.1 ± 3.5 (20.1, 0.1)	<0.001	24.6 ± 3.0	23.2 ± 3.4	<0.001
SBP (mmHg)^b^	109.1 ± 10.6 (108.1, 0.1)	104.4 ± 9.2 (105.3, 0.2)	<0.001	119.2 ± 14.2	110.7 ± 14.1	<0.001
DBP (mmHg)^b^	66.4 ± 9.7 (65.8, 0.1)	65.6 ± 8.4 (66.0, 0.1)	0.304	81.7 ± 10.4	73.6 ± 9.6	<0.001
Hypertension (%)^a^	7.1 ± 2.6 (7.3, 0.1)	4.6 ± 2.1 (4.6, 0.1)	<0.001	23.9 ± 4.3	7.5 ± 2.6	<0.001
Total cholesterol (mg/dl)^a^	157.0 ± 27.3 (155.4, 0.2)	164.4 ± 26.3 (166.9, 0.2)	<0.001	192.9 ± 35.8	190.6 ± 36.1	<0.001
Triglyceride (mg/dl)^a^	155.2 ± 143.4 (152.3, 0.5)	88.2 ± 50.3 (148.8, 0.5)	<0.001	170.1 ± 137.7	110.4 ± 78.9	<0.001
HDL-cholesterol (mg/dl)^a^	50.0 ± 10.0 (47.3, 0.1)	52.4 ± 10.0 (53.7, 0.1)	<0.001	46.4 ± 10.8	53.5 ± 12.0	<0.001
LDL-cholesterol (mg/dl)^a^	90.0 ± 23.6 (90.7, 0.2)	94.4 ± 23.1 (94.5, 0.2)	<0.001	112.5 ± 35.5	115.1 ± 31.9	<0.001
Fasting glucose (mg/dl)^a^	91.3 ± 8.0 (92.9, 0.1)	90.0 ± 9.4 (88.4, 0.1)	<0.001	102.2 ± 24.0	96.0 ± 20.4	<0.001
Energy intake (kcal/day)^a^	2304.8 ± 918.9 (2293.1, 3.9)	1838.9 ± 723.0 (1755.0, 3.9)	<0.001	2420.5 ± 975.1	1701.5 ± 666.6	<0.001
Salt intake (mg/day)^a^	4080.4 ± 2298.5 (4071.9, 12.3)	3171.6 ± 1892.2 (3184.5, 12.3)	<0.001	5323.1 ± 3444.4	3702.2 ± 2551.7	<0.001
Hard exercise (hour/week)^a^	3.5 ± 5.1 (3.5, 0.1)	1.8 ± 4.0 (1.8, 0.1)	<0.001	2.1 ± 5.4	1.3 ± 4.1	<0.001
Medium exercise (hour/week)^a^	3.0 ± 5.2 (3.0, 0.1)	2.2 ± 4.0 (2.2, 0.1)	<0.001	3.3 ± 7.3	2.9 ± 7.2	<0.001
Walking (hour/week)^a^	6.4 ± 7.3 (6.3, 0.1)	5.0 ± 6.8 (5.3, 0.1)	<0.001	6.2 ± 8.6	5.2 ± 6.9	<0.001

Mean ± standard deviation (Adjusted mean, standard error) for boys and girls, and mean ± standard deviation for father and mother

*p value between boys and girls

**p value between father and mother

^a^adjusted for age for boys and girls

^b^adjusted for age and height for boys and girls

Table 2 shows the relationship of BP between offspring and their parents using linear regression analysis. In Model 1, there were positive correlations for SBP and DBP in all of the following comparisons between offspring and their parents: offspring and their mothers (standardized beta coefficients (Sb); 0.185 for SBP, and 0.14 for DBP); offspring and their fathers (Sb; 0.166 for SBP, and 0.118 for DBP); boys and their mothers (Sb; 0.19 for SBP, and 0.133 for DBP); boys and their fathers (Sb: 0.164 for SBP, and 0.139 for DBP); girls and their mothers (Sb: 0.123 for SBP, and 0.143 for DBP); and girls and their fathers (Sb; 0.151 for SBP, and 0.087 for DBP) (all *P* < 0.001). In Model 2, there were also positive correlations for SBP and DBP in all the following comparisons between offspring and their parents: offspring and their mothers (Sb; 0.161 for SBP, and 0.13 for DBP); offspring and their fathers (Sb; 0.15 for SBP, and 0.111 for DBP); boys and their mothers (Sb; 0.176 for SBP, and 0.128 for DBP); boys and their fathers (Sb: 0.148 for SBP, and 0.131 for DBP); girls and their mothers (Sb: 0.135 for SBP, and 0.136 for DBP); and girls and their fathers (Sb; 0.152 for SBP, and 0.081 for DBP) (all *P* < 0.001).

**Table 2:** Linear regression analysis of blood pressure between offspring and parents

			Model 1				Model 2			
Group	Dependent variables	Independent variables	*b*	SE	Sb	*p* value	*b*	SE	Sb	*P* value
All offspring	SBP (z-score)	SBP(mother)	0.011	0.001	0.185	<0.001	0.010	0.001	0.161	<0.001
		SBP(father)	0.010	0.001	0.166	<0.001	0.009	0.001	0.150	<0.001
	DBP (z-score)	DBP(mother)	0.014	0.002	0.140	<0.001	0.013	0.002	0.130	<0.001
		DBP(father)	0.011	0.001	0.118	<0.001	0.011	0.001	0.111	<0.001
boys	SBP (z-score)	SBP(mother)	0.012	0.001	0.190	<0.001	0.011	0.001	0.176	<0.001
		SBP(father)	0.010	0.001	0.164	<0.001	0.009	0.001	0.148	<0.001
	DBP (z-score)	DBP(mother)	0.014	0.002	0.133	<0.001	0.014	0.002	0.128	<0.001
		DBP(father)	0.014	0.002	0.139	<0.001	0.013	0.002	0.131	<0.001
Girls	SBP (z-score)	SBP(mother)	0.008	0.001	0.123	<0.001	0.008	0.001	0.135	<0.001
		SBP(father)	0.009	0.001	0.151	<0.001	0.010	0.001	0.152	<0.001
	DBP (z-score)	DBP(mother)	0.014	0.002	0.143	<0.001	0.013	0.002	0.136	<0.001
		DBP(father)	0.008	0.002	0.087	<0.001	0.007	0.002	0.081	<0.001

*b* beta coefficient, *SE* standard error, *Sb* standardized beta coefficient

Model 1: adjusted for age of offspring

Model 2: adjusted for age, BMI z-score, lipid profile, glucose, physical activity, total calorie intake, and total salt intake of offspring

**Fig. 1:**
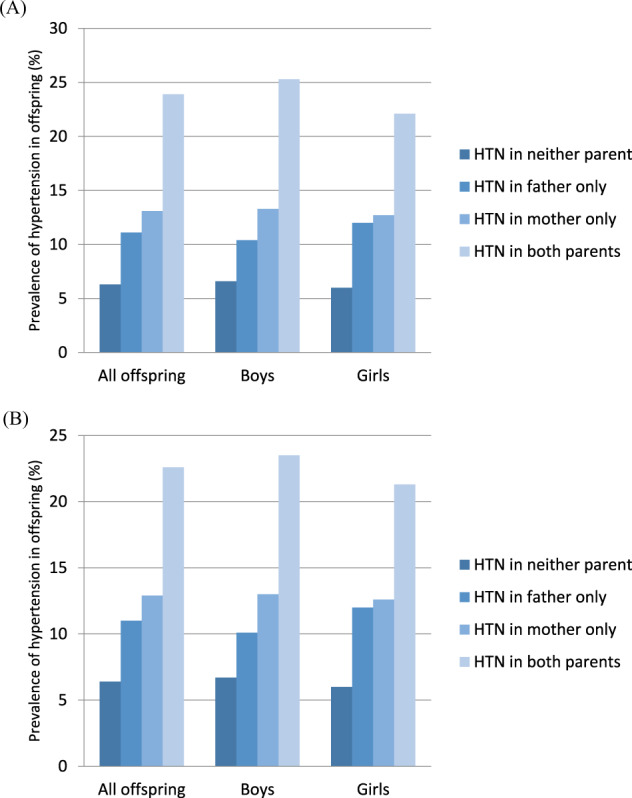
Prevalence of hypertension in offspring based on parental hypertension status adjusted for age (**A**) and adjusted for age, BMI, lipid profile values, glucose levels, physical activity, calorie intake, and salt intake of the offspring (**B**). (HTN; hypertension)

To assess the effect of having hypertensive parents, offspring were divided into four groups: (1) neither parent was hypertensive, (2) only the father was hypertensive, (3) only the mother was hypertensive, and (4) both parents were hypertensive. The prevalence of hypertension in offspring tended to increase based on the number of parents with hypertension (Fig. 1). When adjusted for age (A), the prevalence of hypertension was 6.3%, 11.1%, 13.1%, and 23.9% when neither parent, only the father, only the mother, and both parents had hypertension, respectively. When analyzed separately for boys and girls, the prevalence of hypertension showed patterns similar to those seen in the analysis of all offspring; 6.6%, 10.4%, 13.3%, and 25.3% of boys and 6%, 12%, 12.7%, and 22.1% of girls had hypertension when neither parent, only the father, only the mother, and both parents had hypertension, respectively. When additionally adjusted for BP-influencing factors, including BMI, lipid profile values, glucose levels, physical activity, total calorie intake, and total salt intake (B), the prevalence of hypertension increased with the number of parents with hypertension. For offspring, the prevalence of hypertension was 6.4%, 11%, 12.9%, and 22.6% when neither parent, only the father, only the mother, and both parents had hypertension, respectively. The trend was similar when divided into boys and girls: 6.7%, 10.1%, 13%, and 23.5% for boys and 6%, 12%, 12.6%, and 21.3% for girls.

Table 3 shows the OR of having hypertension among offspring with parental hypertension; the group with parental hypertension was compared with the control group without parental hypertension, adjusting for age in Model 1 and additionally adjusting for BMI, lipid profile values, glucose levels, physical activity, total calorie intake, and total salt intake in Model 2.

**Table 3:** Odds ratio of having hypertension among offspring in the presence of parental hypertension, compared with in the control group where neither parent is hypertensive

			Model 1			Model 2		
Group	Parental hypertension	*N*	OR	95% CI	*p* value	OR	95% CI	*p* value
All offspring	Neither parent (reference)	2837 (180)	–	–	–	–	–	–
	Mother only	205 (26)	2.286	1.470–3.554	<0.001	2.247	1.439–3.510	<0.001
	Father only	861 (97)	1.875	1.446–2.431	<0.001	1.833	1.410–2.384	<0.001
	Both parents	93 (22)	4.823	2.913–7.987	<0.001	4.224	2.522–7.076	<0.001
Boys	Neither parent (reference)	1574 (104)	–	–	–	–	–	–
	Mother only	124 (16)	2.230	1.267–3.923	0.005	2.153	1.214–3.821	0.009
	Father only	474 (50)	1.655	1.161–2.359	0.005	1.595	1.114–2.285	0.011
	Both parents	52 (13)	5.021	2.586–9.748	<0.001	4.154	2.104–8.202	<0.001
Girls	Neither parent (reference)	1263 (76)	–	–	–	–	–	–
	Mother only	81 (10)	2.321	1.143–4.714	0.028	2.345	1.150–4.780	0.019
	Father only	387 (47)	2.169	1.478–3.183	<0.001	2.156	1.465–3.175	<0.001
	Both parents	41 (9)	4.554	2.091–9.920	<0.001	4.247	1.922–9.384	<0.001

*OR* odds ratio, *CI* confidence interval, *N* number of subjects (number of hypertensive subjects)

Model 1: adjusted for age of offspring

Model 2: adjusted for age, BMI z-score, lipid profile, glucose, physical activity, total calorie intake, and total salt intake of offspring

In Model 1, the odds ratio of having hypertension was approximately doubled when either parent had hypertension compared with when neither parent had hypertension; the OR was 2.286 when only the mother had hypertension (*P* < 0.001) and 1.875 when only the father had hypertension (*P* < 0.001). In the group where both parents had hypertension, the risk of hypertension was over four times greater than that in the control group (OR: 4.823, *P* < 0.001). When offspring were divided based on sex, the ORs of having hypertension among the cases versus controls had a similar tendency to that shown in the analysis of all offspring; the ORs of having hypertension were 2.230 (*P* = 0.005) in boys and 2.321 (*P* = 0.028) in girls when only the mother had hypertension, 1.655 (*P* = 0.005) in boys and 2.169 (*P* < 0.001) in girls when only the father had hypertension, and 5.021 (*P* < 0.001) in boys and 4.554 (*P* < 0.001) in girls when both parents had hypertension (Table 3).

In Model 2, the result showed a similar trend to that shown in Model 1. The odds ratios of having hypertension when compared to the control group were as follows: the OR was 2.247 (*P* < 0.001), 1.833 (*P* < 0.001), and 4.224 (*P* < 0.001) for all offspring, 2.153 (*P* = 0.009), 1.595 (*P* = 0.011), and 4.154 (*P* < 0.001) for boys, and 2.345 (*P* = 0.019), 2.156 (*P* < 0.001), and 4.247 (*P* < 0.001) for girls, when only the mother, only the father, and when both parents had hypertension, respectively (Table 3).

**Fig. 2:**
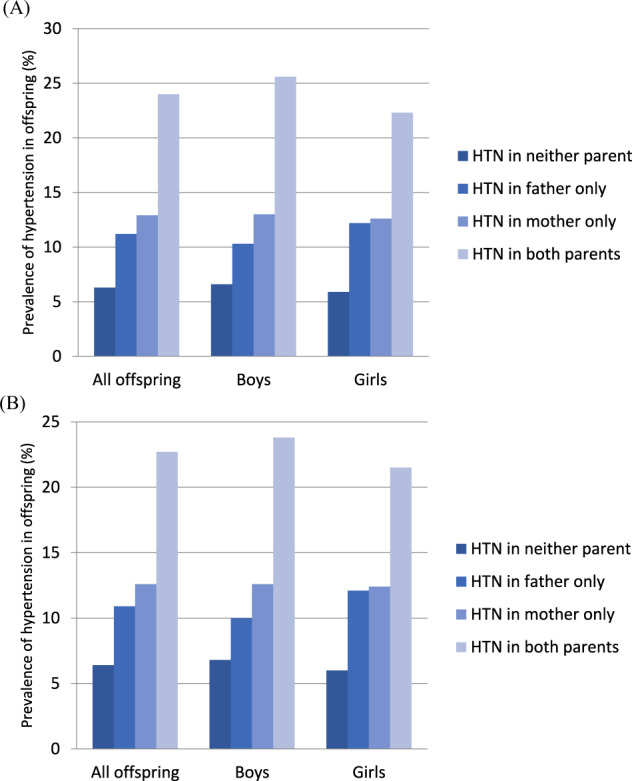
Prevalence of hypertension in offspring aged 10–15 years based on parental hypertension status adjusted for age (**A**) and adjusted for age, BMI, lipid profile values, glucose levels, physical activity, calorie intake, and salt intake of the offspring (**B**). (HTN hypertension)

**Fig. 3:**
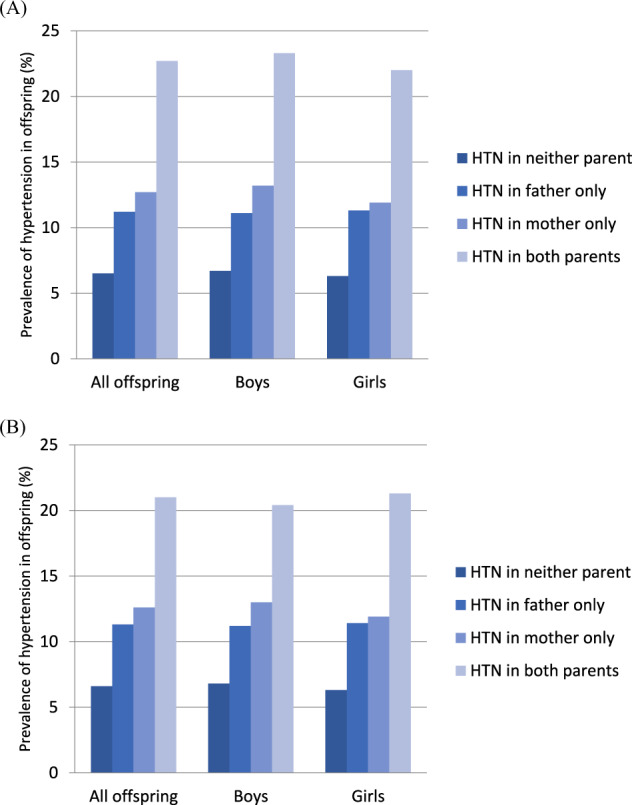
Prevalence of hypertension in offspring aged 16–18 years based on parental hypertension status adjusted for age (**A**) and adjusted for age, BMI, lipid profile values, glucose levels, physical activity, calorie intake, and salt intake of the offspring (**B**). (HTN hypertension)

We analyzed the prevalence of hypertension and the OR of having hypertension among offspring in different age groups separately (aged 10–15 years and aged 16–18 years) (Figs. 2 and 3 and Tables 4 and 5). All the results of these different age groups showed similar trends as those of all age groups.

**Table 4:** Odds ratio of having hypertension among offspring aged 10–15 years in the presence of parental hypertension, compared with in the control group where neither parent is hypertensive

		Model 1				Model 2		
Group	Parental hypertension	*N*	OR	95% CI	*p* value	OR	95% CI	*p* value
All offspring	Neither parent (reference)	2283 (144)						
	Mother only	134 (17)	2.234	1.305–3.827	0.003	2.193	1.271–3.786	0.005
	Father only	702 (79)	1.894	1.419–2.529	<0.001	1.827	1.361–2.451	<0.001
	Both parents	67 (16)	4.877	2.706–8.791	<0.001	4.282	2.328–7.877	<0.001
Boys	Neither parent (reference)	1247 (82)						
	Mother only	86 (11)	2.164	1.104–4.245	0.025	2.089	1.052–4.149	0.035
	Father only	384 (40)	1.652	1.111–2.457	0.013	1.555	1.037–2.333	0.033
	Both parents	35 (9)	4.966	2.249–10.964	<0.001	4.206	1.858–9.522	0.001
Girls	Neither parent (reference)	1036 (62)						
	Mother only	48 (6)	2.313	1.145–5.160	0.006	2.316	1.138–5.115	0.007
	Father only	318 (39)	2.226	1.458–3.397	<0.001	2.197	1.431–3.373	<0.001
	Both parents	32 (7)	4.808	1.983–11.656	0.001	4.383	1.751–10.970	0.002

*OR* odds ratio, *CI* confidence interval, *N* number of subjects (number of hypertensive subjects)

Model 1: adjusted for age of offspring

Model 2: adjusted for age, BMI z-score, lipid profile, glucose, physical activity, total calorie intake, and total salt intake of offspring

**Table 5:** Odds ratio of having hypertension among offspring aged 16–18 years in the presence of parental hypertension, compared with in the control group where neither parent is hypertensive

		Model 1				Model 2		
Group	Parental hypertension	*N*	OR	95% CI	*p* value	OR	95% CI	*p* value
All offspring	Neither parent (reference)	554 (36)						
	Mother only	71 (9)	2.100	1.196–4.070	0.006	2.136	1.173–4.288	0.006
	Father only	159 (18)	1.842	1.215–3.145	0.004	1.893	1.235–3.063	0.004
	Both parents	26 (6)	4.187	1.577–11.113	0.004	3.526	1.302–9.551	0.013
Boys	Neither parent (reference)	327 (22)						
	Mother only	38 (5)	2.125	1.175–3.993	0.015	2.089	1.272–5.072	0.018
	Father only	90 (10)	1.749	1.128–2.848	0.016	1.859	1.183–3.558	0.013
	Both parents	17 (4)	4.196	1.259–13.990	0.020	3.141	1.389–10.231	0.007
Girls	Neither parent (reference)	227 (14)						
	Mother only	33 (4)	2.015	1.106–4.057	0.025	2.062	1.126–4.075	0.023
	Father only	69 (8)	1.923	1.158–3.482	0.016	1.941	1.168–3.588	0.016
	Both parents	9 (2)	4.213	1.792–13.406	0.009	3.931	1.373–11.148	0.011

*OR* odds ratio, *CI* confidence interval, *N* number of subjects (number of hypertensive subjects)

Model 1: adjusted for age of offspring

Model 2: adjusted for age, BMI z-score, lipid profile, glucose, physical activity, total calorie intake, and total salt intake of offspring

## Discussion

In the present study, we investigated the association of BP and hypertension between offspring and their parents. We found that both boys and girls had positive correlations with both parents concerning SBP and DBP. The prevalence of hypertension in boys and girls tended to increase based on the number of parents with hypertension. Additionally, the odds of having hypertension among offspring approximately doubled or quadrupled when either or both parents had hypertension, respectively, compared to offspring without parents with hypertension. Even after adjustment for BP-influencing factors, the results were consistent with those without adjustment, showing that parental BP and hypertension independently affected the BP and hypertension of offspring. When analyzed separately in the different age groups, the prevalence of hypertension and the OR of having hypertension showed similar trends to those described above in all age groups.

To our knowledge, this is the first population-based study in Korea to show the association of hypertension between offspring and their parents separately according to sex. Previous studies have evaluated the familial association of BP and aggregation of hypertension in various populations. A Finnish study of 15-year-old children and their parents reported a statistically significant mother-offspring correlation of SBP, father-offspring correlation of mean arterial pressure (MAP), and a higher proportion of offspring in the highest quartile of SBP and MAP when their mother, but not their father, had a history of hypertension (OR = 3.4, and OR = 2.6, respectively) [7]. A random sample of an Italian nursery and school-age population reported significant correlations in both SBP and DBP between mother and son (*r* = 0.17 and *r* = 0.11), mother and daughter (*r* = 0.11 and *r* = 0.15), and father and son (*r* = 0.16 and *r* = 0.17), but no significant difference in the father–daughter correlations [15]. Friedlander et al. reported that in a sample of Jewish families in Jerusalem consisting of children aged 17–18 years and their parents, mother-child correlation coefficients for SBP (0.16) and DBP (0.15) were higher than those of father‐child correlations (SBP = 0.13; DBP = 0.09) [16]. Additionally, they reported that among four ethnic origin groups (Europe, Asia, North Africa, and Israel), parent‐child correlations for SBP and DBP were highest in the European and Asian groups, lowest in the North African group, and intermediate in the Israeli group [16]. In a case‒control study of American subjects aged 19 years or younger with essential hypertension (EH) and their parents, the odds ratio of having at least one parent with EH was almost 7-fold greater, and the odds of having two parents with EH was 14-fold greater in subjects with EH than in subjects without EH [8]. Li et al. demonstrated that the prevalence rate of hypertension in first-degree relatives was significantly higher (34.44%) than that in second-degree relatives (17.60%) or third-degree relatives (13.51%) in the Han population in Shanghai, China [6].

As shown above, studies from various countries, including Korea, are not entirely consistent. Genetic factors, obesity, and environmental factors, such as nutrition and physical activity, are known to affect BP [17, 18], and these influencing factors may be the reason for the different patterns of familial associations of BP and hypertension seen among other countries and ethnic groups. Therefore, to establish a medical policy regarding identifying and managing hypertension, every country needs to have data on the familial association of BP and hypertension.

### Perspective

From a clinical perspective, hypertension in children has been increasing in many countries, including Korea [19]. Hypertension in childhood increases the risk of cardiovascular diseases in adulthood [10] and even increases the risk of cardiovascular disease for other family members [20]. Therefore, early diagnosis and management of childhood hypertension are needed to decrease mortality and morbidity in adulthood and the overall health care demand of these patients. Furthermore, identifying the risk factors for hypertension in childhood is vital for prevention and early management of this disease. In this context, our findings suggest that parental hypertension could indicate childhood hypertension. As hypertension does not usually manifest as symptoms, especially in children, children identified as being at risk of hypertension can be screened by measuring BP at regular intervals. Applying prevention methods such as reducing childhood obesity, watching one’s diet, and increasing physical activity might be a highly cost-effective medical policy considering the potential advantages [21].

For more targeted prevention and management of hypertension, the identification of hypertension-related genes is needed. Understanding the familial association of disease could be an essential first step in discovering disease-causing genes. Our study shows the clear parent-offspring correlation of hypertension and provides an essential basis for future research on hypertension-causing genes.

### Asian perspective

We aimed to help predict the risk of hypertension among offspring related to parental hypertension in other Asian countries by providing data on the Korean familial association of blood pressure.

### Limitations

There would be a possibility that the subjects underreported their lifestyle, including physical activity and nutrition, when answering the questionnaire. Additionally, as the study used a cross-sectional design, it was impossible to define lifelong relationships.

## Conclusion

We confirmed the close relationship of blood pressure and hypertension between offspring and their parents in South Korea. Further studies are required to investigate the genes causing hypertension.

## References

[1] Li FR, He Y, Yang HL (2021). Isolated systolic and diastolic hypertension by the 2017 American College of Cardiology/American Heart Association guidelines and risk of cardiovascular disease: a large prospective cohort study. J Hypertens.

[2] Wan EYF, Chin WY, Yu EYT (2021). Retrospective cohort study to investigate the 10-year trajectories of disease patterns in patients with hypertension and/or diabetes mellitus on subsequent cardiovascular outcomes and health service utilisation: a study protocol. BMJ Open.

[3] Wang X, Xu X, Su S, Snieder H (2015). Familial aggregation and childhood blood pressure. Curr Hypertens Rep.

[4] Brandao AP, Brandao AA, Araujo EM, Oliveira RC (1992). Familial aggregation of arterial blood pressure and possible genetic influence. Hypertension.

[5] Badaruddoza, Kaur P (2012). Familial aggregation of blood pressure with respect to anthropometric variables among the Lobana (nomadic origin) population in Punjab, India. Asia Pac J Public Health.

[6] Li AL, Fang X, Zhang YY, Peng Q, Yin XH (2019). Familial aggregation and heritability of hypertension in Han population in Shanghai China: a case-control study. Clin Hypertens.

[7] Fuentes RM, Notkola IL, Shemeikka S, Tuomilehto J, Nissinen A (2000). Familial aggregation of blood pressure: a population-based family study in eastern Finland. J Hum Hypertens.

[8] Gupta-Malhotra M, Hashmi SS, Barratt MS, Milewicz DM, Shete S (2018). Familial aggregation of first degree relatives of children with essential hypertension. Blood Press.

[9] Din-Dzietham R, Liu Y, Bielo MV, Shamsa F (2007). High blood pressure trends in children and adolescents in national surveys, 1963 to 2002. Circulation.

[10] Yang L, Magnussen CG, Yang L, Bovet P, Xi B (2020). Elevated blood pressure in childhood or adolescence and cardiovascular outcomes in adulthood: a systematic review. Hypertension.

[11] Prevention KCfDCa. Korea National Health and Nutrition Examination Survey. http://knhanes.kdca.go.kr.

[12] Lurbe E, Agabiti-Rosei E, Cruickshank JK (2016). 2016 European Society of Hypertension guidelines for the management of high blood pressure in children and adolescents. J Hypertens.

[13] Kim SH, Park Y, Song YH (2019). Blood Pressure Reference Values for Normal Weight Korean Children and Adolescents: Data from The Korea National Health and Nutrition Examination Survey 1998-2016: The Korean Working Group of Pediatric Hypertension. Korean. Circ J.

[14] Williams B, Mancia G, Spiering W (2018). 2018 ESC/ESH Guidelines for the management of arterial hypertension: The Task Force for the management of arterial hypertension of the European Society of Cardiology and the European Society of Hypertension: The Task Force for the management of arterial hypertension of the European Society of Cardiology and the European Society of Hypertension. J Hypertens.

[15] Fossali E, Ruzza ML, Codega C (1990). Familial aggregation of blood pressure in a paediatric population. Acta Paediatr Scand.

[16] Friedlander Y, Kark JD (1984). Familial aggregation of blood pressure in a Jewish population sample in Jerusalem among ethnic and religious groupings. Soc Biol.

[17] He FJ, MacGregor GA (2006). Importance of salt in determining blood pressure in children: meta-analysis of controlled trials. Hypertension.

[18] Owen CG, Nightingale CM, Rudnicka AR (2010). Physical activity, obesity and cardiometabolic risk factors in 9- to 10-year-old UK children of white European, South Asian and black African-Caribbean origin: the Child Heart And health Study in England (CHASE). Diabetologia.

[19] Cho H, Kim JH (2020). Secular trends in hypertension and elevated blood pressure among Korean children and adolescents in the Korea National Health and Nutrition Examination Survey 2007-2015. J Clin Hypertens (Greenwich).

[20] Kondo T, Toyoshima H, Tsuzuki Y (2005). Familial aggregation and coaggregation of history of hypertension and stroke. J Hum Hypertens.

[21] Falkner B, Lurbe E (2020). Primordial prevention of high blood pressure in childhood: an opportunity not to be missed. Hypertension.

